# Factors associated to serum 25-hydroxyvitamin D levels among older adult populations in urban and suburban communities in Shanghai, China

**DOI:** 10.1186/s12877-017-0632-z

**Published:** 2017-10-24

**Authors:** Qun Cheng, Yanping Du, Wei Hong, Wenjing Tang, Huilin Li, Minmin Chen, Songbai Zheng

**Affiliations:** 1 Department of Osteoporosis and Bone Disease, Research Section of Geriatric Metabolic Bone Disease, Shanghai Geriatric Institute, Huadong Hospital affiliated to Fudan University, Shanghai, China; 2 Research Center on Aging and Medicine, Fudan University, 221 West Yan An Road, Shanghai, 200040 China

**Keywords:** Vitamin D deficiency, Community, Older adults, Sociodemographic factors, Lifestyle, Dietary factors

## Abstract

**Background:**

Vitamin D deficiency is widespread in China, particularly among older adults. Factors associated with suboptimal vitamin D levels are not well defined. The present study was a population-based study that included 10 urban and suburban communities in Shanghai, to evaluate vitamin D status and its correlates among older adults.

**Method:**

This cross-sectional study was based on study data for 3924 healthy men and women aged 65−95 years. Anthropometric and socioeconomic data were collected in June−July 2014. Serum 25(OH)D levels were detected using a chemiluminescence immunoassay. The following socioeconomic data were obtained through self-administered questionnaires: education level, lifestyle, residency, and dietary habits. A logistic regression model was used to assess associations between anthropometric factors, socioeconomic factors and serum 25(OH)D levels.

**Results:**

Median levels of serum 25(OH)D in men and women were 22.73 and 19.99 ng/mL, respectively. Vitamin D deficiency was common in subjects, even though data collection was conducted during summer. The general prevalence of serum 25(OH)D levels <20 ng/mL were 35.4% and 50.5% for men and women respectively. The general prevalence of serum 25(OH)D levels <10 ng/mL were 2.73% and 5.9% for men and women respectively. A multivariable model indicated serum 25(OH)D levels ≥20 ng/mL were significantly and positively correlated with male sex, calcium or vitamin D supplementation, and residency in suburban communities. The model also indicated that high level of physical activity was protective factors of vitamin D deficiency for men and milk consumption for women, respectively. By contrast, deficient serum 25(OH)D levels were significantly correlated with education level (lower than primary school) or obesity [body mass index (BMI) ≥ 26.06 kg/m^2^] for men or women, respectively.

**Conclusion:**

This cross-sectional study of older adults in communities in Shanghai demonstrates that key factors positively correlated with serum 25(OH)D levels ≥20 ng/mL include male sex, residency in suburban communities, calcium or vitamin D supplementation, high physical activity and education level (for men), and dairy consumption and maintenance of normal BMI (for women).

**Electronic supplementary material:**

The online version of this article (10.1186/s12877-017-0632-z) contains supplementary material, which is available to authorized users.

## Background

Vitamin D deficiency is coming into a global health problem [1], particularly among young children, pregnant women, and older adults. Vitamin D is crucial for bone and mineral homeostasis, particularly for preventing osteomalacia and secondary hyperparathyroidism in older adults. Vitamin D insufficiency is a potential risk factor of osteoporosis, cardiovascular disease, diabetes, cancer, and autoimmune diseases such as rheumatoid arthritis and multiple sclerosis [2–6]. Considering the significance of vitamin D for skeletal and extra-skeletal health, optimal vitamin D level should be maintained especially in older adults.

There is growing concern regarding the high prevalence of hypovitaminosis D in China [7]. However, the causes of widespread vitamin D deficiency among older adults in China are not completely clear. As far as we know, few studies have investigated the factors associated with vitamin D levels among the older adult population in China. Information on participating factors of vitamin D status in older adults may facilitate the development of effective interventions. In this study, we selected 3924 subjects (1677 men and 2247 women) aged 65−95 years to assess potential correlations between vitamin D status and sociodemographic and lifestyle factors. Subjects were selected from a community-based osteoporosis prevention study conducted at Huadong Hospital (affiliated with Fudan University), Shanghai, China, during June and July, 2014. Our three aims were as follows: (i) evaluate vitamin D status in a large cohort of adults older than 65 years who were recruited from urban and suburban areas in Shanghai, East China; (ii) investigate the correlations of serum 25-hydroxyvitamin D [25(OH)D] levels with demographic, lifestyle, physical exercise and dietary factors; and (iii) compare potential differences of serum vitamin D levels in residents of urban and suburban areas.

## Methods

### Study population

The present cross-sectional study was based on study data compiled from healthy male and female subjects in 10 communities in and in Shanghai, China (latitude 31°N; the Shanghai Community Osteoporosis Study), which was conducted from June to July (4 weeks) in 2014. The data consisted mostly of a general health examination and self-reported questionnaires. Health examination data were obtained with standard methods using the same equipment and conducted in a single center.

Subjects recruitment was as previous study [8]. All study subjects were independently ambulant. Subjects who sit in a wheelchair or stayed in bed were excluded from the study. Medical histories and physical examinations confirmed that all participants were healthy old people, without severe diseases interfering with vitamin D metabolism, such as hyperthyroidism, hyperparathyroidism, hepatic failure, renal failure, or end-stage cancer. Subjects with idiopathic bone disease were also excluded from this study. None of the participants were taking any medications that could affect bone or vitamin D metabolism, such as anti-osteoporotic drugs (e.g., glucocorticoids, heparin, warfarin, thyroxine, sex hormones, bisphosphonates, selective estrogen receptor modulators (SERMs), calcitonin, parathyroid hormone (PTH) analogue, or calcitriol). All subjects were recruited by advertisement, and signed the informed consent before study. The study program was approved by the Huadong Hospital Ethics Committee.

A total of 4864 older adults people from communities in and in Shanghai participated in the health study. We implemented the following exclusion criteria: participants younger than 65 years (*n* = 167), participants without a valid serum 25(OH)D measurement (*n* = 102), those not finishing the health examination (*n* = 207), those not completing the questionnaires (*n* = 233), and those who did not give consent to the study (*n* = 231). After these individuals were excluded, a total of 3924 subjects remained for subsequent analysis.

### Data collection

#### Anthropometry

All demographic data were collected and recorded by trained staff. Weight and height were measured without shoes. Body mass index (BMI) was calculated as weight in kilograms divided by the height squared in meters (kg/m^2^).

#### Covariates

Participants completed a self-reported questionnaire (for details, see Additional file 1) on demographic factors, lifestyle factors, and dietary habits. Demographic factors included age, sex, residency, and education level. Lifestyle factors included physical activity, alcohol consumption, and smoking status. Dietary factors included milk consumption and taking calcium or vitamin D supplements.

#### Assessment criteria

We used the following five assessment criteria.

1. Physical exercise was defined as running, walking, dancing, tai chi, and ball games. Housework was not considered a form of physical exercise. High exercise levels were defined as more than 30 min/day or an average of more than 210 min/week. Low exercise levels were defined as less than 30 min/day or an average of less than 210 min/week. No exercise was considered as not performing any of the defined exercises for over 1 year.
2. Current smoker was defined as more than four cigarettes/week within 30 days prior to the health survey. Ex-smoker was defined as previous smoker, but with a current status of no smoking for at least 30 days. Subjects were classified as never smoking if they had no smoking history or if they smoked less than four cigarettes/week within 30 days prior to the health survey.
3. Consumption of ≥3 alcoholic beverages/week was defined as more than 3 times/week, or an average daily amount of alcohol consumption more than 3 units/week for at least 5 years without interruption [per the standardized alcohol consumption index in the Fracture Risk Assessment Tool (FRAX)]. Consumption of fewer than 3 alcoholic beverages/week was defined as less than 3 times/week, or an average daily amount of alcohol consumption less than 3 units/week. No consumption of alcoholic drinks was defined as no history of alcohol.
4. Milk consumption was assessed based on the following three levels: ≥ 250 mL/day; <250 mL/day but ≥50 mL/day; and < 50 mL/day (no milk consumption).
5. Taking vitamin D or calcium supplements was defined as follows: 2800 IU vitamin D/week at least for 1 year; and >400 mg calcium/day at least for 1 year.

### Measurement of serum 25(OH)D levels

Blood samples were obtained from study subjects in June−July 2014. Serum 25(OH)D levels were measured using standard method. Participants were instructed to fast for at least 12 h before analysis. Fasting blood samples were collected in the morning, immediately centrifuged, and the supernatant was transferred to new tubes. The serum samples were sent to Shanghai Key Laboratory of Clinical Geriatric Medicine for analysis within 6 h of blood draw. Serum 25(OH)D levels were measured by performing competitive electrochemiluminescence immunoassays using the Elecsys 2010 system (Roche Diagnostics, US). The intra-assay coefficient of variation (CV) was 2.8%, and the inter-assay CV was approximately 3.0−3.6%. The lower limit of detection was 4 ng/mL.

### Statistical analysis

Statistical analysis was performed by SAS version 19.2. Categorical variables were considered as amounts and percents; continuous variables were considered as median and percentile. Descriptive statistics (frequencies, median, and quartile) were used to evaluate serum 25(OH)D levels. Data normality was assessed by Kolmogorov-Smirnov test. Serum 25(OH)D levels and impact factors were not normally distributed; thus, they were analyzed using a non-parametric test. Differences in gender characteristics and between subjects with serum 25(OH)D levels ≥20 ng/mL or <20 ng/mL were compared using an independent *t*-test for normally distributed data or a Wilcoxon rank sum test for skewed data. Logistic regression models were developed to analyze associations between vitamin D deficiency and possible risk factors. Adjusted odds ratios (ORs) were calculated using serum 25(OH)D < 20 ng/ml as the dependent variable and serum 25(OH)D ≥ 20 ng/mL as the reference category. Univariate analysis was performed to arrive at a final model that included significant (*p* < 0.2) independent variables in the adjusted logistic regression analysis. Differences were considered as statistically significant at *p* < 0.05.

## Results

### Characteristics of the study population

The general characteristics of the study subjects were presented in Table 1. A total of 3924 participants (1677 men and 2247 women) were included in this study. The mean age was 72 years for both genders. Factors significantly differed between male and female, including education level, physical activity, alcohol consumption, cigarette smoking, milk consumption, and supplementation with calcium or vitamin D. By contrast, demographic factors did not significantly differ including age, BMI and residency between men and women. These results suggest that gender-specific differences in vitamin D levels may not be due to demographic factors.

**Table 1:** Descriptive characteristics of the study population ^a)^

		Male		Female		*P* value
		Median	P_25_–P_75_	Median	P_25_–P_75_	
BMI		23.67	21.72–25.91	23.82	21.77–26.06	0.1450
age		72.00	68.00–78.00	72.00	68.00–78.00	0.3835
		*N* (%)		*N* (%)		
Education	Primary education	337 (20.10)		661 (29.42)		< 0.0001
	Middle education	749 (44.66)		896 (39.88)		
	College or above	469 (27.97)		283 (12.59)		
	Under primary	122 (7.27)		407 (18.11)		
Physical exercise	Never	322 (19.20)		541 (24.08)		0.0003
	Low	548 (32.68)		744 (33.11)		
	High	807 (48.12)		962 (42.81)		
Alcohol consumption	Never	1092 (65.12)		2179 (96.97)		< 0.0001
	< 3drink /week	340 (20.27)		60 (2.67)		
	≥ 3drink /week	245 (14.61)		8 (0.36)		
Cigarette smoking	Never	1028 (61.30)		2216 (98.62)		< 0.0001
	Ex-smoker	217 (12.94)		2 (0.09)		
	Current smoker	432 (25.76)		29 (1.29)		
Milk consumption	Never	665 (39.65)		672 (29.91)		< 0.0001
	< 250 ml/day	696 (41.50)		1099 (48.91)		
	≥ 250 ml/day	316 (18.84)		476 (21.18)		
Calcium or vitamin D supplements	No	1461 (87.12)		1770 (78.77)		< 0.0001
	Yes	216 (12.88)		477 (21.23)		
Community	urban	1105 (65.89)		1451 (64.57)		0.3919
	suburb	572 (34.11)		796 (35.43)		

^a)^Data are presented as median, 25th and 75th percentiles for continuous variables and frequencies and percent for categorical variables

### Vitamin D levels in study subjects and the prevalence of vitamin D deficiency in older adults

Serum 25(OH)D levels were relatively skewed for study subjects of both genders; the median and mean vitamin D levels for men were 22.73 and 24.1 ng/mL, respectively, and for women were 19.99 and 21.0 ng/mL, respectively. Approximately 1693 (43.1%) subjects had vitamin D deficiency [25(OH)D concentration < 20 ng/mL], and 1505 (38.4%) had vitamin D insufficiency [25(OH)D concentration of 20−29.9 ng/mL]. The prevalence of vitamin D deficiency and insufficiency in men was 35.5 and 42.0%, respectively; that in women was 48.9 and 35.7%, respectively. Our data show that 4.6% of subjects (2.73% of men and 5.9% of women) had severe vitamin D deficiency [25(OH)D concentration < 10 ng/mL]. Only 17.5% of subjects (22.77% of men and 13.76% of women) had optimal vitamin D levels [25(OH)D concentration ≥ 30 ng/mL).

### Relationships between socioeconomic/sociodemographic factors and vitamin D levels in older adults

The relationships between socioeconomic and sociodemographic factors and serum 25(OH)D levels in older adults are presented in Table 2. Significant differences were observed in serum 25(OH)D levels with respect to age, sex, education level, physical activity, alcohol consumption, cigarette smoking, supplementation with calcium or vitamin D, BMI, and residence in urban or suburban communities. We considered that confounding factors could modify these correlations. Therefore, single factors with significance of *P* < 0.2 were selected for further multi-factor logistic regression analysis.

**Table 2:** Vitamin D status according to different characteristics of the study population

		Vitamin D concentrations			*P* value
		Median	P_25_	P_75_	
Sex	Male	22.73	17.63	28.92	<0.0001
	Female	19.99	15.38	25.62	
Age ^a^	Q1	21.92	16.29	27.55	0.0006
	Q2	21.18	16.39	27.26	
	Q3	21.07	16.44	27.15	
	Q4	20.52	15.24	26.76	
Education level	Primary education	22.17	16.77	29.41	<0.0001
	Middle education	20.67	15.90	26.29	
	College or above	21.04	16.16	26.35	
	Under primary	20.40	15.92	26.47	
Physical exercise	Never	20.01	14.95	27.15	<0.0001
	Low	20.76	16.04	26.25	
	High	21.70	16.78	27.64	
Alcohol consumption	Never	20.64	15.84	26.51	<0.0001
	< 3 drink/week	22.76	17.54	29.74	
	≥ 3 drink/week	24.23	18.86	31.23	
Cigarette smoking	Never	20.66	15.93	26.55	<0.0001
	Ex-smoker	23.16	17.68	29.82	
	Current smoker	22.67	17.06	29.49	
Milk consumption	No	21.16	16.32	27.32	0.1909
	< 250 ml/day	20.74	15.90	26.93	
	≥ 250 ml/day	21.43	16.40	26.80	
Calcium or vitamin D supplements	No	20.59	15.74	26.33	<0.0001
	Yes	23.57	18.20	29.93	
BMI ^b^	Q1	21.42	16.03	28.72	0.0012
	Q2	21.59	16.60	27.25	
	Q3	20.83	16.18	26.75	
	Q4	20.45	15.68	25.69	
Community	urban	20.16	15.44	25.65	<0.0001
	suburb	22.75	17.94	29.77	

^a^ *age: *Q1: < 68 yr.; Q2: 68-71 yr.; Q3: 72-77 yr.; Q4: ≥ 78 yr

^b^ *BMI (Kg/m* ^*2*^ *):* Q1: < 21.76; Q2: 21.76–23.77; Q3: 23.77–25.99; Q4: ≥ 25.99

### Factors associated with vitamin D deficiency

For logistic regression analyses of correlating factors, all subjects were assigned to one of two groups as follows: serum 25(OH)D < 20 ng/mL or 25(OH)D ≥ 20 ng/mL. The regression analyses identified socioeconomic/sociodemographic factors associated with serum 25(OH)D deficiency (Tables 3 and 4). Sex, age, education level, physical exercise, alcohol consumption, smoking, milk consumption, supplementation with calcium or vitamin D, BMI, and residence in urban or suburban communities were included in the logistic regression analysis. Gender-specific lifestyle differences were used to build the statistic models. The results showed that education level and physical activity were significant correlating factors for men, whereas milk consumption and BMI were significant correlating factors for women (Tables 3 and 4). Taking calcium or vitamin D supplements and residence in urban or suburban communities were significant correlating factors for both genders (Tables 3 and 4).

**Table 3:** Logistic regression analysis for vitamin D deficiency by determinants in men

		25(OH)D concentration		*P* value	OR (95% CI)
		< 20 ng/L	≥ 20 ng/L		
		*N*(%)	*N*(%)		
Age^a^	Q1	108(6.55)	211(12.79)	0.5823	1.000
	Q2	173(10.48)	298(18.06)	0.5716	1.128(0.829–1.537)
	Q3	143(8.67)	278(16.85)	0.3979	0.993(0.721–1.368)
	Q4	171(10.36)	268(16.24)	0.2960	1.185(0.853–1.645)
Education level	Primary education	100(6.06)	235(14.24)	0.0059	1.000
	Middle education	251(15.21)	484(29.33)	0.0564	1.157(0.850–1.575)
	College or above	188(11.39)	272(16.48)	0.3294	1.510(1.056–2.158)
	Under primary	56(3.39)	64(3.88)	0.0115	1.980(1.266–3.098)
Physical exercise	Never	138(8.36)	172(10.42)	0.0060	1.000
	Low	197(11.94)	347(21.03)	0.3360	0.712(0.531–0.955)
	High	260(15.75)	536(32.48)	0.0086	0.634(0.479–0.839)
Alcohol consumption	Never	405(24.55)	670(40.61)	0.1102	1.000
	< 3 drink/week	118(7.15)	217(13.15)	0.9510	0.846(0.646–1.107)
	≥ 3 drink/week	72(4.36)	168(10.18)	0.1510	0.728(0.528–1.003)
Cigarette smoking	Never	366(22.18)	646(39.15)	0.3147	1.000
	Ex-smoker	73(4.42)	143(8.67)	0.4040	0.958(0.692–1.326)
	Current smoker	156(9.45)	266(16.12)	0.1350	1.201(0.925–1.559)
Milk consumption	No	230(13.94)	422(25.58)	0.0998	1.000
	< 250 ml/day	265(16.06)	429(26.00)	0.0629	1.095(0.864–1.390)
	≥ 250 ml/day	100(6.06)	204(12.36)	0.0516	0.794(0.582–1.084)
Calcium or vitamin D supplements	No	542(32.85)	893(54.12)		1.000
	Yes	53(3.21)	162(9.82)	<0.0001	0.488(0.347–0.686)
BMI ^b^	Q1	157(9.52)	261(15.82)	0.6327	1.000
	Q2	135(8.18)	275(16.67)	0.1989	0.834(0.621–1.122)
	Q3	153(9.27)	259(15.70)	0.7678	0.966(0.719–1.298)
	Q4	150(9.09)	260(15.76)	0.7410	0.970(0.719–1.307)
Community	urban	425(25.76)	654(39.64)		1.000
	suburb	170(10.30)	401(25.87)	0.0012	0.646(0.496–0.841)

^a^ *Age:* Q1: <68 yr.; Q2: 68–71 yr.; Q3: 72–77 yr.; Q4: ≥ 78 yr

^b^ *BMI (Kg/m* ^*2*^ *): *Q1: <21.72; Q2: 21.72–23.66; Q3: 23.67–25.90; Q4: ≥ 25.91

**Table 4:** Logistic regression analysis for vitamin D deficiency by determinants in women

		25(OH)D concentration		*P* value	OR (95% CI)
		< 20 ng/L	≥ 20 ng/L		
		*N*(%)	*N*(%)		
Age ^a^	Q1	208(9.47)	237(10.79)	0.6197	1.000
	Q2	311(14.16)	320(14.57)	0.9569	1.106(0.859–1.425)
	Q3	291(13.25)	285(12.98)	0.6194	1.154(0.888–1.499)
	Q4	288(13.11)	256(11.66)	0.3855	1.192(0.907–1.567)
Education level	Primary education	285(12.98)	362(16.48)	0.2082	1.000
	Middle education	477(21.72)	393(17.90)	0.2841	1.237(0.975–1.568)
	College or above	142(6.47)	133(6.06)	0.7122	1.097(0.794–1.515)
	Under primary	194(8.83)	210(9.56)	0.3314	1.252(0.965–1.625)
Physical exercise	Never	270(25.96)	243(11.07)	0.4292	1.000
	Low	372(16.94)	364(16.58)	0.5202	0.875(0.689–1.111)
	High	456(20.77)	491(22.36)	0.4029	0.866(0.689–1.089)
Milk consumption	No	341(15.53)	321(14.16)	0.0215	1.000
	< 250 ml/day	542(24.68)	538(24.50)	0.8547	0.845(0.687–1.040)
	≥ 250 ml/day	215(9.80)	239(10.88)	0.0126	0.691(0.532–0.898)
Calcium or vitamin D supplements	No	919(41.85)	812(36.98)		1.000
	Yes	179(8.15)	286(13.02)	< 0.0001	0.443(0.355–0.554)
BMI ^b^	Q1	251(11.43)	303(13.80)	0.0068	1.000
	Q2	268(12.20)	280(12.75)	0.8450	1.178(0.922–1.506)
	Q3	271(12.34%)	278(12.66%)	0.4542	1.129(0.882–1.445)
	Q4	308(14.02%)	237(10.79%)	0.0013	1.539(1.199–1.974)
Community	urban	792(36.07)	610(27.78)		1.000
	suburb	306(13.93)	488(22.22%)	< 0.0001	0.394(0.314–0.493)

^a^ *Age:* Q1: < 68 yr.; Q2: 68–71 yr.; Q3: 72–77 yr.; Q4: ≥ 78 yr

^b^ *BMI (Kg/m* ^*2*^ *):* Q1:< 21.77; Q2: 21.77–23.81; Q3: 23.82–26.05; Q4: ≥ 26.06

For men (Table 3), low education level (uneducated or less than primary school) was a significant risk factor for serum 25(OH)D deficiency (OR >1), whereas physical exercise (duration ≥30 min), calcium or vitamin D supplementation, and residence in suburban community were significant protective factors (OR <1). Vitamin D deficiency was more prevalent among male subjects with low education levels than in those who had at least completed primary school (OR = 1.980, 95% CI 1.266−3.098, *P* = 0.0115). However, there were no differences in vitamin D status among men with primary school, high school, or college degrees (*P* > 0.05). Physical exercise was a significant factor associated with vitamin D status. Male subjects with ≥30 min/day physical exercise had significantly lower rates of vitamin D deficiency than those with no exercise (OR = 0.634, 95% CI 0.479−0.839, *P* = 0.0086); however, there was no significant difference in vitamin D status among subjects with no exercise and those who exercised for <30 min/day. Male subjects who took calcium or vitamin D supplements or lived in suburban communities had significantly lower prevalence of vitamin D deficiency than those who did not take supplements and lived in urban communities (*p* < 0.0001 and *p* = 0.0012, respectively).

For women (Table 4), milk consumption (≥ 250 mL/day), taking calcium or vitamin D supplements, BMI, and residence in suburban communities were significant protective factors against vitamin D deficiency (OR <1, *p* < 0.05). By contrast, BMI ≥ 26.06 kg/m^2^ was a significant risk factor for vitamin D deficiency (OR >1). Older female subjects with BMI ≥ 26.06 kg/m^2^ had higher vitamin D deficiency rates than those with BMI < 21.77 kg/m^2^ (OR = 1.539, 95% CI 1.199−1.974, *P* = 0.0013). Older female subjects who consumed ≥250 mL/day of milk had a lower prevalence of vitamin D deficiency than those who did not drink milk (OR = 0.691, 95% CI 0.532−0.898, *P* = 0.0126). Taking calcium or vitamin D supplements and living in suburban communities were strongly correlated with vitamin D sufficiency (*p* < 0.0001 for both).

## Discussion

We investigated vitamin D status with a large cohort of older adults in urban and suburban communities in Shanghai, and compared these data with an array of socioeconomic/sociodemographic factors. Our analyses indicate that vitamin D deficiency is highly prevalent among the older adult Chinese population. More than 82.5% of our subjects had serum 25(OH)D concentrations less than 30 ng/mL, and approximately 43% had less than 20 ng/mL, even during summer. Our linear regression analyses identified several potentially modifiable and gender-mediated factors that were independently associated with vitamin D deficiency. For men, low education level (below primary school) and low levels of physical activity (< 30 min/day) were risk factors for vitamin D deficiency. For women, low milk consumption (< 250 mL/day) and high BMI were risk factors for vitamin D deficiency. Residence in suburban communities and taking calcium or vitamin D supplements were protective factors for subjects of both sexes.

### Comparisons of vitamin D deficiency among older adults and other populations in China

Our results on vitamin D deficiency of the older adults in Shanghai during summer were slightly lower than those reported previously for the same population and city during winter. The prevalence of vitamin D insufficiency (< 30 ng/mL) and vitamin D deficiency (< 20 ng/mL) previously reported by Zhang et al. [9] was 84% and 30% in males, and 89% and 46% in females. In northeast China, vitamin D deficiency (< 25 nmol/L) was observed in 48% and 15% of old men and women in early spring [10]. In central China, vitamin D sufficiency [serum 25(OH)D ≧ 75 nmol/L] was observed in 19.4% and 14.4% of men and women aged 60−89 years, respectively [11]. A detailed multicenter study of healthy adults aged >18 years reported the following levels of vitamin D status in Chinese population: 5.9% had vitamin D severe deficiency (< 10 ng/mL), 50.0% had vitamin D deficiency (10−20 ng/mL), 38.7% had vitamin D insufficiency (20−30 ng/mL), and 5.4% had vitamin D sufficiency (> 30 ng/mL) [12].

Currently, there is a lack of standardization among studies investigating vitamin D levels with respect to the method of vitamin D measurement, and the definition of vitamin D deficiency and sufficiency, and seasonal variations. Other studies report that average serum 25(OH)D levels were lowest in winter and highest in summer [13, 14], and the prevalence rate of 25(OH)D deficiency was 4−5 times higher in winter-spring than that in summer-fall [15]. In our population, approximately 33% of men and 50% of women had deficient 25(OH)D concentrations (< 20 ng/mL) in the summer, so we speculate that deficiency rates may be higher in other seasons. Consequently, it is likely that a large proportion of the older adults in Shanghai communities have suboptimal vitamin D concentrations, even during summer, when vitamin D levels should peak.

### Low milk consumption and high BMI were risk factors for vitamin D deficiency in women

Food nutrients and nutritional supplements are known predictors of serum 25(OH)D levels [16, 17]. By comparison, few studies conducted in China have investigated the effects of dairy consumption or nutritional supplements on 25(OH)D levels. Our results indicate that milk consumption is more important in women than in men for vitamin D status, and milk consumption has a significant protective action against vitamin D deficiency in women when >250 mL per day is consumed. A recent study among 504 older Singaporean Chinese adults also reported that 25(OH)D levels were associated with the vitamin D, calcium, and dairy product intake for women but not for men [18]. Sex-mediated differences in dairy consumption and vitamin D status need to be clarified in future investigations.

Our study identified an inverse correlation between serum 25(OH)D levels and higher BMI for women but not for men. Older women with BMI ≥ 26.06 kg/m^2^ had 1.539 times greater vitamin D deficiency rates than those with BMI < 21.77 kg/m^2^. These results were consistent with those of a previous study [19], which also showed that BMI was independent predictors of blood 25(OH)D levels. Other studies in U.S. in immigrant populations reported that BMI is positively correlated with vitamin D deficiency, with a prevalence rate of 62.5% when BMI ≥ 25 vs 56.2% when BMI <25 (*P* = 0.02); however, this research included both men and women but did not differentiate effects of BMI by sex [20]. However, women have more body fat per unit weight than men [21], whereas vitamin D can be stored in fat, which may affect serum 25(OH)D levels. Other mechanism which can affect serum vitamin D levels should be lacking of outdoor activity in obese individuals [22, 23].

### Lack of physical exercise and low education level were risk factors of vitamin D deficiency in men

Our results indicate that >30 min/day of physical exercise confers a significant protective effect against vitamin D deficiency in men, but not in women. A lack of regular physical activity (< 30 min/day) was associated with vitamin D deficiency. Sociodemographic factors may influence this correlation. In China, older women traditionally perform all housework, and they enjoy evening activities such as walking and dancing; however, older men enjoy performing activities such as tai chi and jogging in the morning. Abboud et al. [24] reported that specific uptake of 25(OH)D into muscle may contribute to the long half-life of 25(OH)D in blood, and exercise may extend the half-life of serum 25(OH)D. A recent study [25] also showed that physical activity was a significant positive determinant (β = 0.13) of serum vitamin D levels and smoking was found to be a significant negative determinant (β = −2.64), the latter of which stands in contrast to the present study, which found no such correlation.

Our regression analyses also indicated that a lower education level (those who did not complete primary school) was a significant risk factor for vitamin D deficiency in older men. This result is consistent with that of a previous study, which showed that vitamin D deficiency was common among men with no education and a sedentary lifestyle [26]; however, another study from Southern China found that highly educated single females tend to be vitamin D deficient, which may be affected by a desire to be pale and sun avoidance [27]. We evaluated whether this correlation might be due to confounding factors (for example, men with low socioeconomic status may be prone to alcohol consumption and smoking); however, our analyses did not find any associations with potential confounding factors. Future studies will be required to clarify the observed correlation between low education and vitamin D deficiency among older men.

### Calcium or vitamin D supplementation and residence in suburban communities were predictors of vitamin D sufficiency for older men and women

We identified an association between serum 25(OH)D concentration and resident location (urban or suburban). To our knowledge, this has not been investigated in previous studies conducted in China. We found that older residents in suburban communities had a lower prevalence of vitamin D deficiency than those in urban communities. This may be due to lifestyle differences, such as the amount of outdoor activity (higher in suburban communities) and the use of sun protection tools (higher in urban communities).

Regarding calcium or vitamin D supplements, our results indicate that subjects who take calcium or vitamin D supplements have significantly higher 25(OH)D levels than those who do not. Chan et al. [28] also reported that serum 25(OH)D concentration was associated with vitamin D supplementation rather than dietary habit. A study from Brolsma EM et al. [29] investigated determinants of vitamin deficiency in an older population, and found that sun exposure, genetic background and vitamin D intake were main factors explaining 35% of 25(OH)D status. Brolsma et al. also asserted that vitamin D supplement may be an inexpensive, easy and effective strategy to prevent vitamin D deficiency.

Vitamin D supplements can enhance vitamin D levels, and calcium supplements may facilitate vitamin D utilization. Low levels of dietary calcium intake may give rise to mild hyperparathyroidism and accelerate the conversion of 25(OH)D into 1,25(OH)2D, which consequently reduces 25(OH)D levels [30]. Our results indicate that low vitamin D supplement intake (< 400 IU/day) did not significantly affect vitamin D status; however, intake of ≥ 400 IU/day vitamin D supplement maintained serum 25(OH)D concentration > 20 ng/mL. Few foods naturally contain vitamin D, and no foods are fortified with vitamin D in China; therefore, older adults in China must depend on taking nutritional supplements to maintain vitamin D sufficiency. In our study, taking either calcium or vitamin D supplements had a protective effect against vitamin D deficiency, provided the average calcium or vitamin D intake was > 400 mg or > 400 IU per day, respectively.

### Sub-group analyses

#### Age

There were no significant associations between age and serum 25(OH)D concentrations in our study cohort (aged > 65 years). Previous studies reported decreased cutaneous production of vitamin D with age [31, 32]. Our study did not observe significant differences in serum 25(OH)D levels in people 65−95 years old for either sex, suggesting that age is not a correlating factor within this population. Further studies are required to explore possible stratifying effects of age among older adults.

#### Gender

Our results showed that old women tended to have lower serum 25(OH)D levels than those of old men, which is consistent with other studies [33, 34]. Data from these studies indicate that, compared to women, men had higher 25(OH)D levels regardless of BMI, which is apparently due to the greater body surface area of men that can absorb sunlight. Furthermore, men work outdoors more often.

## Conclusions

Vitamin D deficiency is high prevalent in older adults in China, and many modifiable lifestyle factors are potentially important determinant on vitamin D deficiency. This study showed that taking calcium or vitamin D supplements and living in suburban communities were predictors of vitamin D sufficiency for both sexes. For women, low levels of milk consumption and high BMI were significantly correlated with vitamin D deficiency. For men, low education levels and low physical activity were significantly correlated with vitamin D deficiency. Further studies are wanted on potential correlation mechanisms and optimal strategies to improve vitamin D status in community old population. The results indicated that community old adults should be encouraged to consume more foods with vitamin D, take calcium and vitamin D supplements, drink more milk, and participate in physical exercise to improve their health.

## Additional files

Additional file 1: Bone health questionnaire. (DOCX 18 kb)

## Abbreviations

25(OH)D: 25-hydroxyvitamin D
BMI: Body mass index
CV: Coefficient of variation
ECL: Electrochemiluminescence
FRAX: Fracture Risk Assessment Tool
LC/MS-MS: Liquid chromatography-tandem mass spectrometry
OR: Odds ratios
PTH: Parathyroid hormone
SERMs: Selective estrogen receptor modulators
