# Supplementary material for: Factors associated to serum 25-hydroxyvitamin D levels among older adult populations in urban and suburban communities in Shanghai, China
Source: BMC Geriatr. 2017 Oct 24;17:246. doi: 10.1186/s12877-017-0632-z (PMC5654067; doi:10.1186/s12877-017-0632-z)
Supplement: Supplementary file 1 — Bone health questionnaire. (DOCX 18 kb) [file 12877_2017_632_MOESM1_ESM.docx]

Bone health questionnaire

Name

Visiting date

Telephone number

Home address

Community

Height cm

Weight kg

Birthday

1. Sex ：① male ② female
2. Race ：① Han ② Others
3. Education： ①primary school ②high school ③college or above ④under primary
4. Outdoor exercise ：① no ② yes（＜30 min/d） ③ yes（≥30 min/d）
5. Exercise mode ：

（1）Walk ① no ② seldom ③<3 times/week ④≥3~5 times/week ⑤ everyday

（2）Run ① no ② seldom ③<3 times/week ④≥3~5 times/week ⑤ everyday

（3）Ball game ① no ② seldom ③<3 times/week ④≥3~5 times/week ⑤everyday

（4）Aerobics ① no ② seldom ③<3 times/week ④≥3~5 times/week ⑤everyday

1. Drink ① no ②<3drink /week ③≥3drink /week
2. Smoking ① never ②Ex-smoker，time of start smoking time of quit smoking

③ Current smoker <20 cigarettes/d ④ Current smoker ≥20 cigarettes/d

1. Daily dairy products ①no ②<250ml ③≥250ml
2. vegetarian： ①yes ②no
3. calcium supplement ① no ②<400mg/d, how long ③≥400mg/d, how long
4. Vitamin D supplement ① no ②<2800U/week, how long ③≥2800U/week, how long
